# p53-SOAT1轴：肿瘤相关脂质代谢调控与治疗新靶点

**DOI:** 10.3779/j.issn.1009-3419.2025.106.34

**Published:** 2025-12-20

**Authors:** Xi YU, Yuting XIAO, Bowen XU, Xinhui LI, Hongmei WANG, Dunqiang REN

**Affiliations:** 266000 青岛，青岛大学附属医院呼吸与危重症医学科; Department of Respiratory and Critical Care Medicine, The Affiliated Hospital of Qingdao University, Qingdao 266000, China

**Keywords:** p53, 脂质代谢, SOAT1, 肿瘤, p53, Lipid metabolism, SOAT1, Tumor

## Abstract

癌症治疗是全球医学面临的重大挑战，其复杂的分子机制和药物耐药性是制约治疗效果的关键因素。异常的脂质代谢是肿瘤的重要特征之一，为肿瘤细胞的生长、增殖、迁移和侵袭提供代谢支持。肿瘤抑制因子p53蛋白和甾醇O-酰基转移酶1（sterol O-acyltransferase 1, SOAT1）在调控细胞脂质代谢中发挥重要作用，与多种肿瘤的发生发展及预后密切相关。p53蛋白通过多条信号通路调控肿瘤脂质代谢，而SOAT1作为胆固醇酯化的关键酶，在多种肿瘤中高水平表达并加速肿瘤进展进程。有研究表明p53蛋白与SOAT1可能存在功能性关联作用，协同调控肿瘤细胞的脂质稳态。本文综述了p53蛋白与SOAT1在肿瘤脂质代谢中的研究进展，重点阐述p53-SOAT1轴在肿瘤发生发展中的潜在作用机制，并展望其作为癌症治疗靶点的应用前景。

## Abstract (en)

Cancer treatment is a significant challenge facing global medicine, with complex molecular mechanisms and drug resistance being key factors limiting treatment outcomes. Abnormal lipid metabolism is one of the important characteristics of tumors, providing metabolic support for the growth, proliferation, migration, and invasion of tumor cells. Tumor suppressor p53 protein and sterol O-acyltransferase 1 (SOAT1) play an important role in regulating cellular lipid metabolism and are closely related to the occurrence, development, and prognosis of various tumors. p53 protein regulates tumor lipid metabolism through multiple signaling pathways, while SOAT1, as a key enzyme in cholesterol esterification, is highly expressed in many tumors and accelerates tumor progression. Recent studies have shown that there may be a functional association between p53 protein and SOAT1, coordinating the regulation of lipid homeostasis in tumor cells. This article reviews the research progress on p53 protein and SOAT1 in tumor lipid metabolism, focusing on the potential mechanisms of action of the p53-SOAT1 axis in tumor development, and prospects its application prospects as a target for cancer treatment.

癌症是严重威胁人类健康的疾病，根据GLOBOCAN 2022全球癌症统计数据及人口统计学预测，到2050年全球新发病例将增加至3,528.1万例，比2022年增加76.6%^[1]^。尽管现代医学取得了显著进展，但当前癌症整体预后仍不理想，病死率持续居于高位，亟需基于新分子机制（如代谢轴调控）的治疗策略。异常肿瘤代谢是癌症的标志之一，脂质代谢异常是其中的重要表现^[2-7]^。脂质不仅是生物膜的基本成分和疏水屏障，还作为能量来源和信号分子，支持肿瘤细胞增殖和肿瘤生长^[8,9]^，如高CD36表达通过增加脂肪酸（fatty acid, FA）摄取促进肿瘤细胞生长、迁移，与乳腺癌、卵巢癌、胃癌和前列腺癌患者的预后不良有关^[10]^。胆固醇酯化增强则通过耗竭膜游离胆固醇、破坏脂筏结构而下调主要组织相容性复合体I（major histocompatibility complex-I, MCH-I）分子表达，削弱CD8^+^ T细胞识别，导致免疫逃逸^[11,12]^。*TP53*是位于17号染色体短臂上的肿瘤抑制基因^[13]^，编码p53蛋白。p53蛋白是一种含393个氨基酸的核磷蛋白，在癌症中p53蛋白能抑制细胞增殖，以应对多种刺激，包括DNA损伤、营养剥夺、缺氧、增殖过度信号，从而防止肿瘤形成^[14]^。人类肿瘤中，*TP53*基因功能失活的发生率约为50%^[15]^。当*TP53*基因发生突变时，其肿瘤抑制功能丧失，导致癌细胞不受控制的生长和分裂，在一些恶性肿瘤中*TP53*基因突变与更严重的恶性肿瘤表型有关^[16]^，研究^[17]^表明*TP53*突变可能通过调控脂质代谢促进肿瘤进展。甾醇O-酰基转移酶1（sterol O-acyltransferase 1, SOAT1）是一种位于内质网内的酯化酶，催化游离胆固醇生成胆固醇酯，并储存于脂滴^[18]^。高表达的SOAT1与多种肿瘤进展有关，如肺癌、前列腺癌^[19,20]^。近年来，有研究^[21]^表明SOAT1是许多癌症类型中的潜在药物靶点，SOAT1开始在癌症领域获得高度关注。

越来越多的证据提示p53与SOAT1在肿瘤细胞中通过复杂相互作用协同调控脂质代谢，影响肿瘤生物学行为。深入研究p53-SOAT1轴在肿瘤发生与发展中的作用机制，对揭示肿瘤代谢重编程网络及开发有效癌症治疗策略具有重要意义。本文系统综述p53和SOAT1在肿瘤脂质代谢中的研究进展，重点探讨p53-SOAT1轴的潜在机制及其治疗前景。

## 1 *TP53*突变与脂质代谢的分子机制

p53蛋白是一种重要的肿瘤抑制因子^[22]^，作为一种关键转录因子，p53蛋白通过直接或间接机制调控脂质代谢相关基因的表达，在FA和胆固醇代谢中发挥多种作用。

### 1.1 p53蛋白调控的信号通路

p53蛋白通过调控多种下游靶点显著影响肿瘤细胞代谢。在脂质代谢方面，p53-硬脂酰辅酶A去饱和酶1（stearoyl-CoA desaturase 1, SCD1）轴通过下调SCD1的表达水平，可有效降低单不饱和脂肪酸的合成，影响细胞膜流动性和肿瘤信号转导^[23]^。p53-TREM2轴可能通过调控巨噬细胞中的脂质稳态参与肿瘤微环境重塑^[24]^。在子宫内膜癌中p53蛋白过表达可逆转丝氨酸β-内酰胺酶样蛋白（serine beta-lactamase-like protein, LACTB）沉默诱导恶性表型和脂质代谢重编程效应，提示p53可能通过调控LACTB下游通路抑制脂质异常积累^[25]^。此外，p53蛋白通过激活肉毒碱O-辛酰基转移酶（carnitine O-octanoyltransferase, CROT）和丙二酰辅酶A脱羧酶（malonyl-CoA decarboxylase, MCD），促进不同长度的FA转运到线粒体中进行降解，减少脂质异常积累^[26-28]^。类似地，p53蛋白靶基因*Lpin1*也促进脂肪酸氧化（FA oxidation, FAO）和营养应激条件下的细胞存活^[29]^。肿瘤细胞快速增殖高度依赖FA从头合成途径，因此p53通过抑制脂肪生成、促进脂肪分解及脂质氧化等多重机制限制肿瘤生长^[30]^。

值得注意的是，有研究^[25]^证实p53-SREBP1轴在前列腺癌中通过解除对固醇调节元件结合蛋白-1（sterol regulatory element-binding protein-1, SREBP-1）的抑制，促进FA合成和肿瘤增殖、迁移。而野生型p53蛋白转录抑制SREBP-1，导致脂质抗氧化剂的减少、脂质合成减少^[8]^。同样，*TP53*缺失上调*SREBP-1*及其下游基因表达，而p53蛋白过表达抑制了*SREBP-1c*基因及其下游基因在*TP53*缺失的Saos2骨肉瘤细胞中的启动子活性^[31]^。除此以外，p53蛋白还通过调控甲羟戊酸途径（mevalonate pathway, MVP）影响胆固醇代谢，该途径是胆固醇合成的关键步骤。p53蛋白能够抑制SREBP-2的激活过程，从而下调SOAT1的表达，导致胰腺癌中的MVP活性降低，最终抑制胆固醇合成并限制肿瘤进展^[32]^，这些研究揭示了p53蛋白调控脂质代谢进而影响肿瘤发展的普遍机制，通过抑制脂质合成，从而限制肿瘤发展。

### 1.2 p53蛋白与代谢酶的直接作用

p53蛋白通过与脂质代谢关键酶的直接相互作用调控脂质合成与分解，例如，p53蛋白可结合并抑制葡萄糖-6-磷酸脱氢酶（glucose-6-phosphate dehydrogenase, G6PD），从而降低FA合成所必需的NADPH的水平，进而减少FA的从头合成^[33]^。在胆固醇代谢调控方面，p53蛋白表现出复杂的双向调控特征：一方面，p53蛋白可通过抑制SREBP-2的成熟及下游MVP相关酶（如SOAT1）的表达，从整体上阻断胆固醇的合成与酯化，从而发挥抑癌作用；另一方面，尽管有研究^[34]^发现在特定细胞背景（如人胶质母细胞瘤细胞和正常星形胶质细胞）下，p53蛋白可以通过诱导一组相关酶包括甲羟戊酸激酶（mevalonate kinase, MVK）等的表达，促进MVP的上游活性，但这种作用受到下游反馈机制（如抑制SREBP-2）的严格限制，以避免脂质异常积累。此外，p53蛋白通过上调FAO肉碱棕榈酰转移酶1A（carnitine palmitoyl transferase 1A, CPT1A）的表达，促进脂质分解代谢^[8]^。值得注意的是，癌症基因组图谱（The Cancer Genome Altas, TCGA）数据库及多项队列研究^[34,35]^显示在*TP53*基因突变型肺腺癌中*SREBP1*、*SCD1*、*FASN*等脂质合成基因显著上调，并与更差的总生存期独立关联。

## 2 *SOAT1*在脂质代谢中的重要作用

*SOAT1*是一种编码胆固醇酰基转移酶的基因，通过将游离胆固醇转化为胆固醇酯促进脂滴形成，在多种恶性肿瘤中呈现显著高表达，并促进肿瘤细胞的增殖、侵袭和迁移能力^[35,36]^，如肺癌^[37]^、胃癌^[35]^、口腔鳞状细胞癌^[38]^、前列腺癌^[39]^与胶质母细胞瘤^[40]^、胰腺癌^[41]^，涉及miR-100-3p/SOAT1、血管内皮生长因子（vascular endothelial growth factor, VEGF）-C、SREBP-1/SREBP-2、磷脂酰肌醇3-激酶/蛋白激酶B（phosphatidylinositol 3-kinase/protein kinase B, PI3K/AKT）等多条信号通路。SOAT1在肿瘤脂质代谢重编程的核心作用主要体现在SOAT1快速酯化过量游离胆固醇，避免其引起的膜硬化、内质网应激及凋亡，保护肿瘤细胞^[42]^；此外，SOAT1还通过上调线粒体碎片化和激活信号通路（如PI3K/AKT），促进肿瘤细胞的侵袭^[43]^。研究^[44]^发现，抑制SOAT1可导致游离胆固醇积累，激活LXR、上调MHC-I表达、增强CD8⁺ T细胞抗肿瘤活性，并降低27-羟基胆固醇等免疫抑制代谢产物，从而将“冷肿瘤”转化为“热肿瘤”。以上研究充分揭示了SOAT1在肿瘤脂质代谢重编程和肿瘤进展中的重要作用，提示SOAT1是极具潜力的治疗靶点。

鉴于SOAT1在多种癌症中高表达并与肿瘤进展和不良预后有关，靶向SOAT1已被视为一种有前景的癌症治疗策略，目前越来越多靶向SOAT1抑制肿瘤生长增殖的研究证据出现（表1^[35,42,45-50]^）。有报道^[51]^提示，在*TP53*基因突变肿瘤中SOAT1高表达及脂滴负荷可作为预测抑制剂敏感性的潜在生物标志物。阿伐麦布是第一种也是最常用的SOAT1抑制剂^[52,53]^。临床前研究^[54]^已证明其安全性。在胃癌研究^[35]^中阿伐麦布抑制SOAT1活性可抑制胃癌细胞增殖、胆固醇酯合成和淋巴管生成，其机制是通过抑制SOAT1表达，调控SREBP-1、SREBP-2的表达，降低VEGF-C表达，从而减少淋巴管生成。此外，在结肠癌研究^[47]^中阿伐麦布在两种小鼠模型中均显示出显著疗效：氧化偶氮甲烷/葡聚糖硫酸钠（azoxymethane/dextran sulfate sodium, AOM/DSS）诱导模型和ApcMin/+基因突变模型。有研究^[35,42]^还探索了阿伐麦布与其他药物的联合治疗作用。阿伐麦布和依托莫西在体外和体内对肝细胞癌（hepatocellular carcinoma, HCC）具有协同抗癌作用，其机制是SOAT1抑制导致胆固醇酯化减少，积累游离FA；同时CPT1A抑制阻断这些FA向线粒体转运氧化，防止其转化为能量或通过SOAT1重新酯化为脂滴储存，从而共同破坏肿瘤脂质稳态，诱导细胞毒性。阿伐麦布与制霉菌素通过提高肠癌细胞中胆固醇含量促进Yes相关蛋白1（Yes-associated protein 1, YAP1）表达，在体外和体内协同抑制结肠癌细胞的生存^[42]^。除了阿伐麦布以外，ATR-101也是一种SOAT1特异性抑制剂，已在临床试验中对肾上腺皮质癌（adrenocortical carcinoma, ACC）进行了测试，这项研究^[50]^证明了ATR-101安全性良好，但对晚期ACC缓解率低（<5%-30%），最常见不良反应为可逆性肝酶水平升高以及胃肠道不适症状。诺卡酮（一种倍半萜酮类化合物）可通过靶向SOAT1抑制肿瘤细胞的上皮-间质转化（epithelial-mesenchymal transition, EMT）过程^[18]^，从而抑制HCC发生。此外，有研究^[55]^还筛选了3种化合物：尼洛替尼、ABT-737和依塞曲匹（Evacetrapib），它们表现出与SOAT1的最佳结合和抑制活性。特别是，临床上使用的第二代酪氨酸激酶抑制剂尼洛替尼，显示出对SOAT1的高亲和力蛋白，并通过重编程肿瘤细胞内胆固醇代谢和增强CD8^+^ T细胞和中性粒细胞的作用，在体内和体外显著抑制肿瘤活性。基于以上研究证据，尽管SOAT1单药治疗疗效有限，但联合其他治疗（如免疫治疗）仍具有明确临床转化潜力。

**表 1:** 靶向SOAT1抑制肿瘤的实验与临床证据总结

Medicine	Type of tumor	Phase of study	Research conclusions	SOAT1 specificity	Ref.
Nilotinib	HCC	In vitro cell/ Animal models	Reengineering cholesterol metabolism and enhancing CD8^+^ T cell anti-tumor activity	No (Mainly TKI)	^[42,45]^
Avasimibe	GC, COAD, HCC	In vitro cell/tumor-bearing mouse model	Inhibiting cholesteryl ester accumulation and inducing apoptosis to reduce tumor volume	Yes	^[35,46,47]^
Mitotane	ACC	Clinical use (Standard of care)	Inhibiting SOAT1 and inducing tumor cell apoptosis	Yes	^[48,49]^
ATR-101	ACC	Clinical phase I (NCT01898715)	Evaluating safety and MTD with a 27% SD rate in advanced ACC patients	Yes	^[50]^

HCC: hepatocellular carcinoma; TKI: tyrosine kinase inhibitor; GC: gastric cancer; COAD: colon adenocarcinoma; ACC: adrenocortical carcinoma; SOAT1: sterol O-acyltransferase 1; MTD: maximum tolerated dose; SD: stable disease.

## 3 p53-SOAT1轴在癌症发生发展中的作用

近年来，p53蛋白作为转录因子对SOAT1的调控作用及其对肿瘤脂质代谢的影响在多种癌症类型中得到实验验证，其核心特征是野生型与突变型p53蛋白对SOAT1转录活性存在相反作用。野生型p53蛋白作为经典转录抑制因子，可以直接结合*SOAT1*基因的启动子，抑制SOAT1转录。p53蛋白功能缺失（loss-of-function, LOF）主要表现为转录抑制解除，而功能获得性突变（gain-of-function, GOF）可激活SREBP-2并上调SOAT1表达^[56]^，此过程可能涉及其对共激活因子p300/CBP的招募^[57]^。同样，在动物模型中发现野生型p53蛋白通过阻断SOAT1功能，从而限制胆固醇酯化，而*TP53*突变解除这一抑制^[32]^，促进肿瘤细胞的代谢重编程。相反，*TP53*基因热点突变常导致p53蛋白构象改变并获得新功能，丧失对SOAT1的转录抑制。例如，在胰腺导管腺癌（pancreatic ductal adenocarcinoma, PDAC）模型中，*TP53*/R270C杂合性缺失（loss of heterozygosity, LOH）显著上调SOAT1的表达。其调控过程依赖于突变型p53蛋白的稳定化与SOAT1上游增强子激活增强^[32]^。以上证据表明，p53-SOAT1轴的失调不仅是转录抑制解除的被动过程，更涉及突变型p53蛋白主动上调SOAT1的功能获得性机制。

值得注意的是，目前上述直接因果关系主要在PDAC和HCC中得到较充分验证，而在肺癌等其他肿瘤中的证据仍较为有限。鉴于肺癌中*TP53*约52%的高突变率^[58]^，本课题组推测p53-SOAT1轴可能同样参与肺癌的脂质代谢重编程（图1）：在野生型*TP53*基因存在时，p53蛋白对SOAT1表达具有一定的抑制作用，维持细胞内胆固醇稳态；而当*TP53*发生突变（尤其是一些GOF）或缺失时，解除这种抑制，甚至通过其他途径促进SOAT1的表达，导致胆固醇酯异常积累，从而驱动肿瘤细胞的代谢重编程，支持肿瘤的快速生长和恶性进展。p53-SOAT1轴不仅驱动肿瘤代谢重编程，还通过重塑肿瘤微环境抑制抗肿瘤免疫监视，影响肿瘤免疫逃逸。有研究^[45]^表明，野生型p53蛋白通过直接结合*SOAT1*基因的启动子，抑制SOAT1表达，维持细胞内游离胆固醇稳态，支持MHC-I类分子在肿瘤细胞表面的正确定位和抗原呈递。*TP53*基因突变或缺失导致SOAT1上调，使肿瘤细胞大量将游离胆固醇酯化为胆固醇酯，从而耗竭细胞膜脂筏结构，干扰MHC-I复合物的组装和表面表达，这一过程最终导致细胞毒性T淋巴细胞识别缺陷和免疫监视失效（图2）。在*TP53*缺失HCC模型中，通过抑制SOAT1表达从而激活CD8^+ ^T细胞增殖和干扰素-γ（interferon-γ, IFN-γ）分泌，并与免疫检查点抑制剂联用产生协同作用，显著增强抗肿瘤效果^[45]^。若在肺癌中得以验证，则针对该轴的干预[如SOAT1抑制剂联合程序性死亡蛋白1/程序性死亡配体1（programmed cell death protein 1/programmed death ligand 1, PD-1/PD-L1）抑制剂，或与恢复p53蛋白功能的小分子联合]有望成为*TP53*突变型肺癌的新策略。SOAT1抑制剂（如阿伐麦布）相关临床前研究^[32,42]^已在肝癌和胰腺癌中显示出优于单药的疗效，为肺癌领域的后续探索提供了重要参考。未来需通过肺癌组织队列验证*TP53*突变与SOAT1表达的相关性，并开展针对p53-SOAT1轴的临床试验，以明确其在肺癌中的真实贡献与治疗价值。

**图 1:**
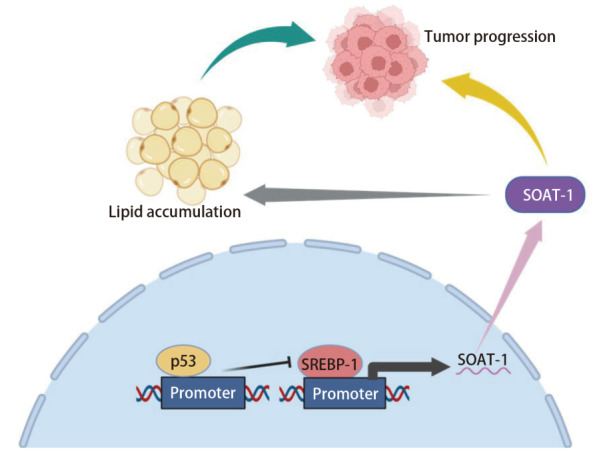
p53-SOAT1轴参与肺癌的脂质代谢重编程机制（本图用BioRender绘制）。当TP53发生突变时，解除对SOAT1的抑制，或通过其他机制上调SOAT1表达（如上调SREBP-1），导致胆固醇酯积累，促进肿瘤进展。

**图 2:**
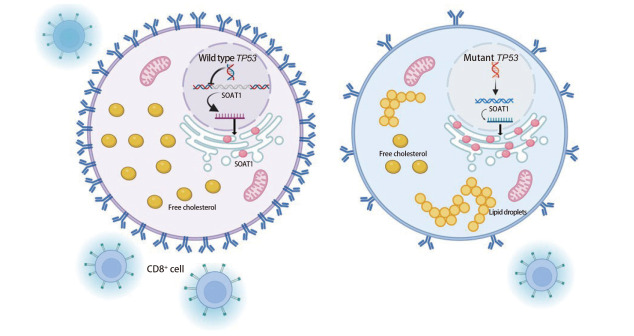
p53-SOAT1轴在肿瘤微环境中的重要作用（本图用BioRender绘制）。野生型p53蛋白直接结合SOAT1基因启动子，抑制SOAT1转录，导致游离胆固醇增多，保护脂筏结构，促进CD8^+^ T细胞介导的肿瘤免疫。突变p53蛋白促进SOAT1表达，导致胆固醇酯增加，破坏脂筏结构，导致免疫逃逸。

## 4 小结与展望

本综述聚焦于p53蛋白、SOAT1相互作用调节脂质代谢在肿瘤发展中的作用，系统阐述了*TP53*基因与SOAT1在肿瘤中的相互作用关系及其对脂质代谢的影响。*TP53*基因通过信号通路、酶的相互作用直接或间接调节肿瘤的脂质代谢。其中SOAT1是脂质代谢的重要组成成分，与多种恶性肿瘤的发生发展有关，如胃癌、前列腺癌、肺癌。在动物模型中野生型p53蛋白通过抑制SOAT1表达，进而抑制肿瘤发展，p53蛋白与SOAT1在肿瘤脂质代谢中都发挥重要作用并以此来影响肿瘤的发展，如*TP53 *LOH显著上调SAOT1、p53蛋白，直接结合SOAT1启动子，从而影响其表达。

基于以上证据，本课题组提出p53-SOAT1轴作为肿瘤代谢重编程的重要调控机制。该轴在胰腺癌、肝癌等癌症中的作用已获初步验证，但在肺癌等*TP53*高突变率肿瘤中的具体机制尚需深入研究，靶向p53-SOAT1轴有望成为肺癌等肿瘤精准治疗的新策略。
